# A perspective on the enabling technologies of explainable AI-based industrial packetized energy management

**DOI:** 10.1016/j.isci.2023.108415

**Published:** 2023-11-11

**Authors:** Daniel Gutierrez-Rojas, Arun Narayanan, Cássia R. Santos Nunes Almeida, Gustavo M. Almeida, Diana Pfau, Yu Tian, Xu Yang, Alex Jung, Pedro H.J. Nardelli

**Affiliations:** 1 School of Energy Systems, Lappeenranta–Lahti University of Technology, Yliopistonkatu 34, 53850 Lappeenranta, South Karelia, Finland; 2 Department of Electrical Engineering, Federal Center for Technological Education of Minas Gerais, Av. Amazonas 5253, Belo Horizonte, MG 30.421-169, Brazil; 3 Department of Chemical Engineering, School of Engineering, Federal University of Minas Gerais, Av. Amazonas 5253, Belo Horizonte, MG 30.421-169, Brazil; 4 Department of Computer Science, Aalto University, Konemiehentie 2, 02150 Espoo, Uusima, Finland

**Keywords:** Energy resources, Energy management, Energy Modeling

## Abstract

This paper reviews the key information and communication technologies that are necessary to build an effective industrial energy management system considering the intermittence of renewable sources like wind and solar ^†^. In particular, we first introduce the concept of software-defined energy networks in the context of industrial cyber-physical systems aiming at the optimal energy resource allocation in terms of its environmental impact. The task is formulated as a dynamic scheduling problem where supply and demand must match at minute-level timescale, also considering energy storage units. The use of (explainable and trustworthy) artificial intelligence (AI), (informative) networked data, demand-side management, machine-type (wireless) communications, and energy-aware scheduling in industrial plants are explored in detail. The paper also provides a framework for understanding the complexities of managing renewable energy sources in industrial plants while maintaining efficiency and environmental sustainability.

## Graphical abstract

**Figure:**
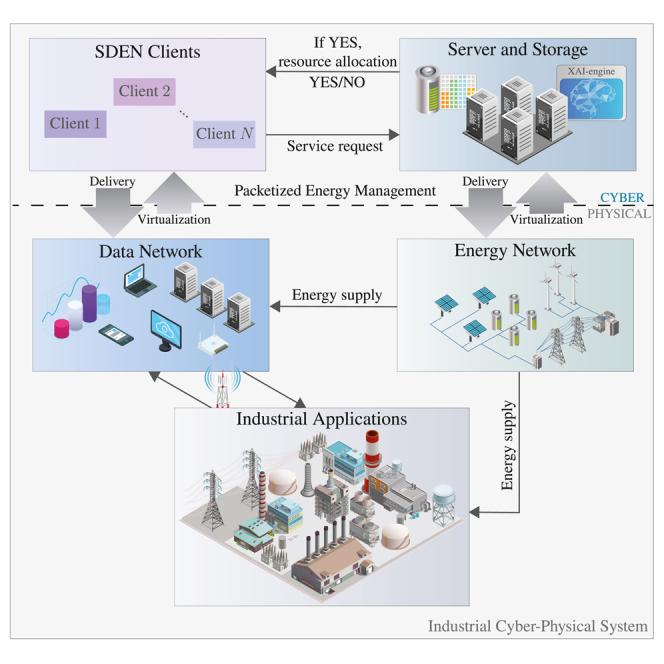

## Teaser

Energy resources; Energy management; Energy Modelling

## Introduction

The latest reports published by Intergovernmental Panel on Climate Change on the impact of climate change (in early 2022) point to the enormous challenges ahead of us (Pörtner et al.).^1^ Decreasing fossil fuel consumption is a necessary condition for a sustainable future. However, despite our awareness about the dangers of this accumulation during the last 30 years and our desire to reduce fossil fuel usage, the statistics show a clear contradiction: fossil fuel consumption has steadily increased (Pirani).^2^ This indicates that the demand for energy continues to grow, and hence, there is an even greater urgency to improve the efficiency and reliability of energy networks while reducing their carbon footprint. In response to this challenge, the research community, media, and policy makers are radically transforming the energy sector, driven by technological advancements. However, their main focus so far seems to be residential and transportation emissions, which directly affect end users and residential customers. On the other hand, the industrial sector requires urgent attention for energy management and control, since it has the largest share of the energy used in most countries. As indicated by the U.S. Energy Information Administration (Source: https://www.eia.gov/), the industrial share of the energy consumed in the USA in 2021 was 33%, which includes facilities and equipment used for manufacturing, agriculture, mining, and construction.

Some previous approaches have focused on industrial demand-side management (DSM), for example, in Lindberg et al.^3^ where a use case of a company’s production is given along with their possibilities and limitations. In Helin et al.,^4^ a DSM case in pulp and paper industry is presented. In this approach, the economic potential of using DSM in mechanical pulp production is emphasized with a mathematical model to assess the technical costs. Another interesting use case is described in Valdes et al.^5^ The authors presented a statistical study using time series analysis and demand profile clustering for industrial consumers. Their results showed flexibility potential to cut peak demand during winter times, and discussed how these approaches are not being in use due to non-existing incentives to carry DSM.

In this paper, we examine the area of industrial energy management, focusing on managing the schedules of industrial processes in a flexible and energy-efficient manner. For this purpose, we propose that the new and emerging paradigm of software-defined energy networks (SDEN) can be employed. SDEN leverages packetized energy management (PEM) to provide more flexible, reliable, and sustainable energy services in the industrial sector, where it has the potential to enhance the efficiency of energy-intensive processes. We explore the intersection of renewable energy and industrial energy management with DSM, focusing on how SDEN via PEM can help integrate renewable energy sources into the grid and manage their variability. In particular, we delve into the technical aspects of PEM and SDEN, including the role of software-defined networking and the Internet of Things in enabling more dynamic and responsive energy networks. We discuss the challenges and opportunities associated with implementing SDEN via PEM in industrial settings and highlight some of the key technologies and strategies that can facilitate this transition. We also discuss the emerging field of machine-type communications and its potential to enable new applications of SDEN via PEM, particularly in the context of mobile edge computing and beyond-5G networks. We highlight some of the key research directions and challenges in this area and discuss how they can shape the future of SDEN via PEM.

With the rapid growth of networked data and the increasing reliance on artificial intelligence (AI) and machine learning (ML) in energy systems, the need for explainability and trustworthiness in energy networks has become more critical than ever. Hence, we explore the concept of explainable and trustworthy AI-based networked sensing for energy packets, which aims to provide transparent and interpretable insights into energy data and ensure the trustworthiness of AI and ML models used in energy systems. We begin by examining the role of AI and ML in energy systems and the challenges they pose for explainability and trustworthiness. We discuss how AI and ML models can be used to analyze networked data from sensors and provide valuable insights into energy consumption patterns, fault detection, and predictive maintenance. Further, we explain the problem that since these models often operate as black boxes, it is difficult to understand their decisions and trust their outputs. Next, we delve into the concept of networked sensing for energy packets, which involves the use of sensors to monitor and collect data on energy consumption and production. We discuss how networked sensing can provide real-time data on energy usage, enabling more effective energy management and optimization, while at the same time, making it challenging to extract meaningful insights by generating a huge amount of data. Finally, we explore the concept of explainability in energy networks, and, in particular, we describe the importance of ensuring the trustworthiness of energy data, including data provenance and data quality assurance.

Finally, we propose a pathway to develop effective energy management of industrial production plants that rely on renewable energy sources such as large-scale solar photovoltaic (PV) parks, wind farms, or hydro power plants. The energy supply from these sources can match the demand in average terms, but this is not possible on operational timescales. To address this challenge, the paper adapts the SDEN introduced in Nardelli et al.^6^ by adding new specifications to the SDEN clients, which in the industrial case collaborate to jointly perform tasks. These can be, and normally are, sequential. Consequently, the proposed SDEN for industrial energy management has more challenging requirements compared to residential SDEN, because the feasible operational plans of the different clients are dependent, and thus, more constrained.

**Figure 1:**
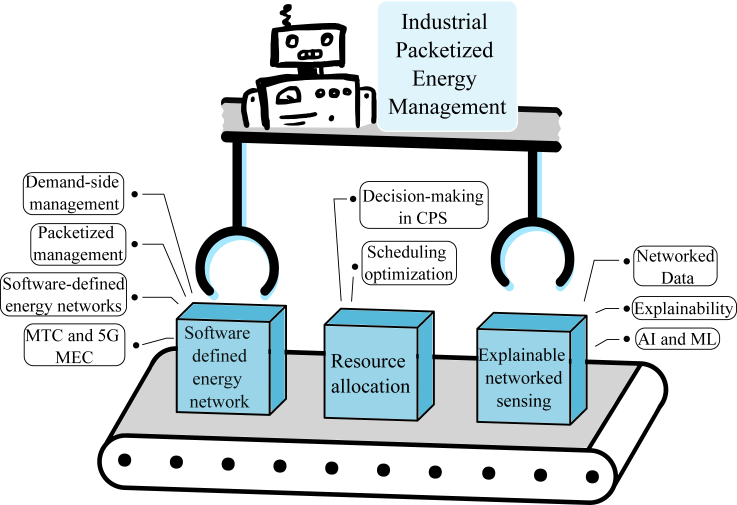
Overview of enabling technologies for industrial packetized management

Figure 1, as a whole, illustrates a smart and interconnected approach to industrial packetized energy management. It emphasizes the importance of using software-defined networks to control energy distribution, efficient resource allocation, and the use of networked sensing for real-time data collection and analysis. This integrated approach allows for agile and data-driven energy management in industrial settings, ultimately contributing to cost savings, reduced energy waste, and improved overall operational efficiency. These 3 blocks seen in Figure 1 will be further explained in the following sections.

Also, we demonstrate a virtualization process in which we build an SDEN that is tailored for industrial plants based on packetized energy management approach as a way to multiplex different types of industrial energy demand in order to match supply and demand in operational timescales; this solution can be considered to be a specific case of DSM that is enabled by the advances of machine-type communications. Our proposed approach aims to solve a variation of the multi-objective (energy-aware) flexible job-shop scheduling optimization problem with a focus on matching supply and demand of electricity, considering the specifics of the sequential nature of the industrial process (Wang et al.^7^; Mota et al.)^8^ in a distributed manner. This is in contrast to the typical centralized approach based on non-linear optimization or metaheuristics that typically use a static or a probabilistic generated dataset. We also show how a flexible job-shop scheduling optimization problem could be solved in this distributed manner through collective learning, whereas the explainable AI engine that will power the SDEN will be implemented in the future.

The contributions of this scientific paper are summarized as follows.

(1) Reformulation of the SDEN initial proposal to enable energy-centric scheduling and operations of the processes in an industrial plant.
(2) Description of three enabling technologies for AI-based industrial packetized management—software-defined energy network via packetized energy management, explainable trustworthy AI-based networked sensing for energy packetization, and energy resource allocation. An overview of these enabling technologies can be seen in Figure 1.
(3) Demonstration of the packetized energy management approach via a software-defined energy network to perform DSM for scheduling industrial loads to follow renewable energy supply.
(4) Demonstration of the potential to solve job-shop scheduling problems in industrial processes using a decentralized distributed collective learning approach.

The rest of this paper is organized as follows. First, it is described the virtualization process required to build the proposed SDEN. Then, we explain the AI needed for the SDEN networked sensing system that is the main element of the cyber domain of the proposed SDEN, focusing on networked data, explainability, and trustworthiness. Subsequently, the paper illustrates the potential of the proposed solution using a selected relevant study case, the industrial manufacturing setup given in Karimi et al.^9^; Karimi and Kwon.^10^ Finally, it is listed the main conclusions of this work and discusses the future perspectives of this research in the field of information and communication technologies (ICTs) for sustainability.

## Software-defined energy network via packetized energy management

SDEN represents a cutting-edge approach to energy management in various domains, including industrial, commercial, and residential settings like DSM. SDEN leverages advanced software and networking capabilities to optimize energy distribution and consumption. SDEN correlates to resource allocation in three aspects: real-data integration, dynamic allocation, and optimization. Moreover, SDEN and explainable networking sensing are in tune with transparency and accountability. In the following subsections, a more detailed description of SDEN built up in PEM is presented.

### Demand-side management

**Figure 2:**
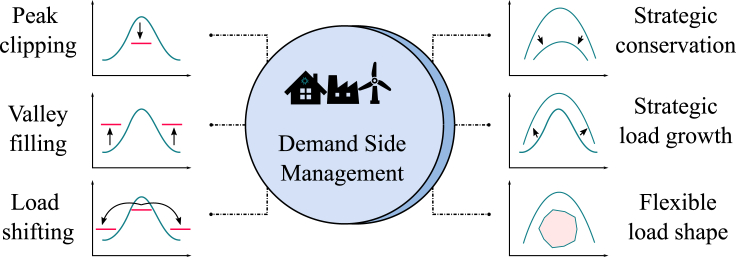
Role of DSM strategies Adapted from Gellings.^12^

DSM is a portfolio of measures to improve the energy system at the consumption side, ranging from improving energy efficiency by using better materials to implementing smart energy tariffs with incentives for certain consumption patterns to sophisticated real-time control of distributed energy resources (Palensky and Dietrich.).^11^ In the case of renewable energy supply, DSM systems aim to control flexible loads to make the demand profile follow the availability of renewable electricity supply, which are less controllable. These sources are usually locally distributed units whose generation fluctuates heavily according to the weather conditions. The local generation reduces the dependence on a centralized energy system. Matching local generation and consumption also reduces the need for the distribution of energy over large distances. The major objectives of DSM are summarized in Figure 2 and they are detailed in the following section (Gellings).^12^

(1) Peak Clipping or Peak Shaving: To reduce demand during peak load periods;
(2) Valley Filling: To increase load during off-peak hours;
(3) Load Shifting: To reschedule the load to off-peak hours or to meet renewable production;
(4) Strategic Conservation: To reduce the energy demand by utilizing more energy efficient appliances;
(5) Strategic Load Growth: To promote applications requiring electricity, e.g., electric vehicles;
(6) Flexible Load Shape: To induce changes in consumption patterns by providing incentives to consumers.

A well-implemented combination of these techniques enables the load shape to follow the generation as close as possible. The overall goal of DSM is to promote the efficient use of energy resources and reduce the pressure on the electrical grid, ultimately reducing costs and promoting sustainability. DSM is a broad term that is associated with different timescales, from primary control at the sub-level to load/storage scheduling plans with time horizons of hours, days, and even months (in the case of renewable energy, with long-term storage). Furthermore, it is also applicable to different types of consumers, offering possibilities for large-scale industries, commercial buildings, and households (Nardelli et al.).^6^

In this paper, we use a software tool called *DEMKit* (Hoogsteen et al.)^13^ to exemplify how a DSM system can work in practice for residential settings. *DEMKit* stands for Decentralized Energy Management Simulation and Demonstration Toolkit, and it was developed at the University of Twente for research on smart grid technologies. Its focus is on the decentralized management of multi-energy systems (electricity, heat, hydrogen, etc.) using a model predictive control system. The control systems are able to optimize local energy usage and thereby reduce the stress on energy distribution networks. *DEMKit* simulates microgrids in residential environments with a wide variety of assets, devices, and technologies, such as photovoltaic panels, batteries, electric cars, dishwashers, washing machines, etc. A modular and cyber-physical systems design paradigm is central to the architecture of *DEMKit*. The system components are modeled individually and they interact and influence each other through predefined interfaces for the different classes. Due to the modular setup, different control systems and device models can be simulated and the effects of control systems on a physical grid can also be evaluated. *DEMKit* is used together with an Artificial Load Profile Generator (ALPG) to form a tool chain for smart grid studies for households and its devices. ALPG creates power profiles of households and devices with explicit information on available flexibility.

**Figure 3:**
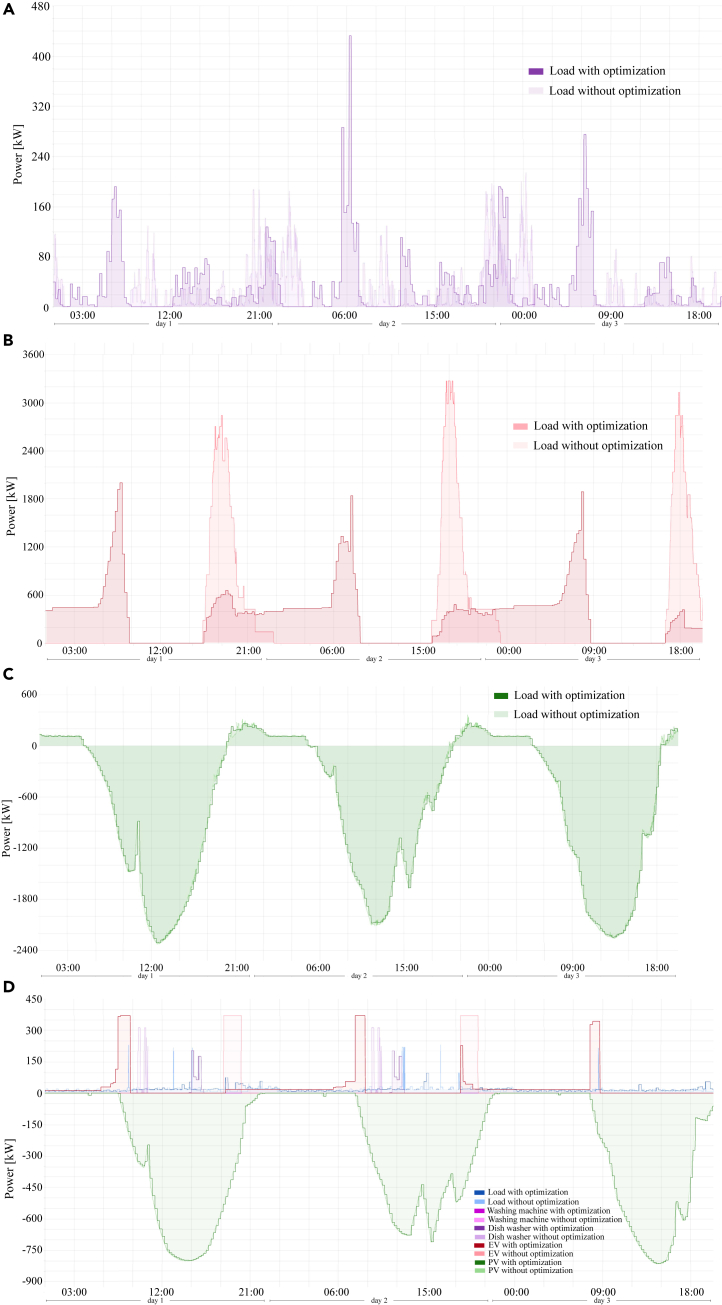
Power profiles and generation before and after being simulated at DEMKit (A) Sum of all time-shiftable in the street for 50 houses. (B) Sum of all buffer-time-shiftable devices in the street for 50 houses. (C) Sum of all curtailable devices in the street for 50 houses. (D) Flexible loads of one house in the street for 50 houses.

As an example, a generic scenario of 50 houses in a street in the Kumpula neighborhood, Helsinki, Finland, was simulated using *DEMKit* and the results are presented in Figure 3. For the ALPG power profiles generation, each house was assumed to have devices with both uncontrollable and controllable loads such as washing machine, dishwasher, electric vehicle, a heat pump, battery, and PV panels. The temperature and the global solar radiation in Kumpula from 04/06/2022 to 06/06/2022 were obtained from the Finnish Meteorological Institute website (Kaurola).^14^

(1) Sum of all time-shiftable devices in the street for 50 houses.
(2) Sum of all buffer-time-shiftable devices in the street for 50 houses.
(3) Sum of all curtailable devices in the street for 50 houses.
(4) Flexible loads of one house in the street for 50 houses.

For the simulation, this set of 50 houses was randomly generated as a mix of homes with one person, a couple, families with a single parent with kids, or both parents with children. The working relationship for each adult varied from unemployed, partial time worker, full time worker, to retired. The household devices and equipment used in each home were also varied. The load profiles were generated by the ALPG and additional electrification and local microgeneration using PV panels were added. The optimization goal for the considered scenario was set to perform peak shaving at the transformer level to reduce or avoid overloading the local grid. Figure 3 shows the power profiles after the application of DSM control strategies (Figure 2) by *DEMKit*. The lightly colored lines are power profiles of different devices without control and the darker lines are the power profiles of the same devices after the control had been applied. In Figure 3A, the power profile sum of all time-shiftable devices of the street (e.g., washing machines or dishwashers) without and with applying optimization is shown. Figure 3B presents the sum of all buffer-time-shiftable devices in the street, e.g., electric vehicles (buffer device with time limitation). Figure 3C shows the sum of all static load and generation that can be curtailed/shed before and after the controls are been applied. In Figure 3D, one single house was chosen to present some of its equipment’s behavior in a more detailed manner; these results are also presented without and with control.

### Industrial demand-side management and renewable energy

Currently, around 80% of the industrial energy needs are met by fossil fuels (EIA),^15^ which are known to be responsible for greenhouse gas (GHG) emissions, mainly carbon dioxide (CO_2_). Compounding this problem, continued world population growth (UN)^16^ will continue to increase industry energy needs. Considering that the industrial sector accounts for more than half of global energy consumption (EIA),^15^ it has a big role in reducing GHG emissions. Oil and gas, steel, cement, pulp and paper, ceramics, glass, and food and beverages are the most representative energy-intensive industries. These processes require heat at relatively very high temperatures, which nowadays are mainly provided by fossil fuels. For example, iron and steel, refineries, cement, and petrochemicals were responsible for almost 70% of the total CO_2_ emissions in the European Union in 2018 (EP).^17^

Environmental restrictions on fossil fuel sources such as pollution and climate change have led to a search for sustainable energy sources. Sustainability basically involves low carbon emissions and greater energy efficiency, which can be achieved by increased use of renewable energy sources. Currently, renewable energy use in industry accounts for just about 10% of total energy use (EIA).^15^ The European Union roadmap targets 80%–95% decarbonization by 2050 compared to 1990 (EC).^18^ Switching from fossil fuels to renewable can be beneficial in many ways. In addition to reducing the use of fossil fuels and therefore greenhouse gas emissions, it also implies better waste management and social sustainability.

Challenges with the wider use of renewable energy sources in energy-intensive processes are primarily related to availability and scaling up. For example, solar and wind power are periodic and variable in nature. Therefore, a major issue for the use of renewable energy concerns meeting the continuous and high demand for energy from industrial processes. An initial approach to resolve this problem is to use the generated electricity for fixed-period operations. A practical example of this case is the paper industry that has a wide range of products. As a first step, the available green electricity can be used to power a part of the production, and conventional electricity can be used for the remainder. This energy packetization approach gives rise to a resource allocation problem, which, in turn, is related to the concept of DSM, explained previously (Zhang and Grossmann).^19^

This opens many possibilities regarding energy forecasting, energy supply planning, energy demand analysis, and, more broadly, energy usage optimization. In addition to decarbonization, such flexibility of electricity use in energy-intensive industries can be seen as a milestone that can be enabled by the advanced ICTs (Ullah et al).^20^ Large-scale industrial applications of DSM can be expected in the medium and long term.

### Industrial packetized energy management and software-defined energy networks

The idea of using discretized energy packets, similar in spirit to data packets transmitted over the internet, is not new. Back in 1996, Saitoh and Toyoda introduced the concepts of open electric energy network and packet electric power transportation (Saitoh and Toyoda).^21^ In recent contributions (Ma et al.),^22^^,^^23^ the authors proposed a distribution grid with local area packetized power networks. Rezaei et al.^24^ describe a decentralized packetized approach to manage electric vehicle charging. Almassalkhi et al.^25^ present a real-time management of thermostatically controlled loads that fulfills the grid needs and requires no specific knowledge about the state of the loads. This proposed scheme is known as packetized energy management (Espinosa and Almassalkhi),^26^ where different loads request energy packets to an energy server, which may grant, schedule, or not grant these requests. This solution is already being commercialized through a start-up company called Packetized Energy (https://arpa-e.energy.gov/technologies/scaleup-launch-pad-2020/packetized-energy). In Espinosa et al.,^27^ the authors present a control-theoretic analysis of their PEM including virtual batteries. We recently provided a more complete literature review in Nardelli et al.^28^ In a series of recent papers (de Castro Tomé et al.^29^; Nardelli et al.^6^; Hussain and Nardelli^30^; Hussain et al.^31^^,^^32^^,^^33^; Mashlakov et al.),^34^ a variation of the cyber-physical packetized energy could be deployed based on simple rules to manage the energy demand of flexible residential loads focusing on the concept of Energy Internet (Hussain et al.)^35^ and commons-based governance model (Giotitsas et al.).^36^^,^^37^

**Figure 4:**
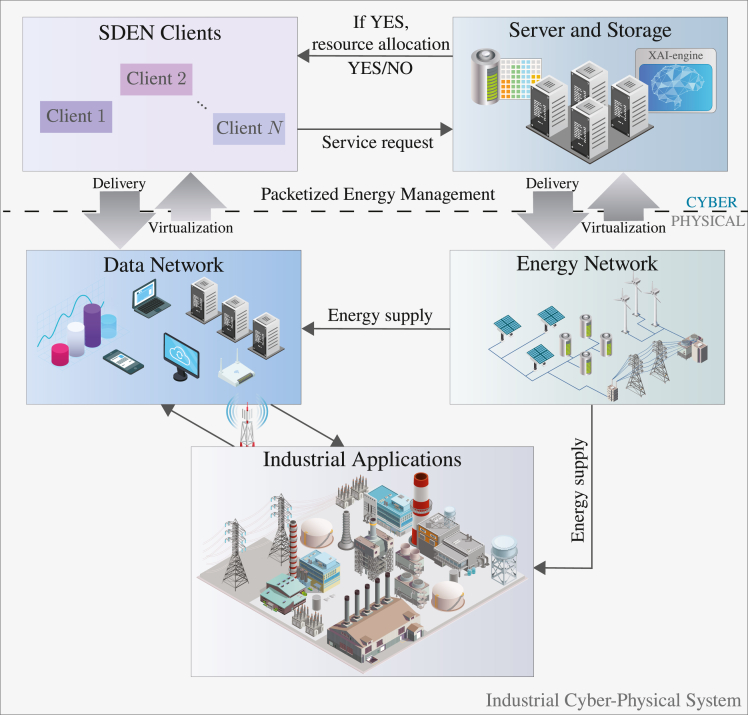
Schematic of the proposed industrial cyber-physical system

In the context of industrial setups, the characterization in Nardelli^38^ states that cyber-physical systems are constituted by three layers—physical, data, and decision—and by three cross-layer processes—sensing, informing, and acting. As a self-developing reflexive-active system, a cyber-physical system is capable of measuring the attributes of the physical system, forming potentially informative data that can then be processed as part of decision-making processes that will guide interventions/actions. The SDEN concept first appeared in Nardelli et al.^6^ as a cyber-physical implementation of a potentially self-sufficient virtual microgrid that is composed of residential electricity consumers. The main idea is to employ a variation of the PEM to multiplex, in the scheduling phase, different types of energy loads, which would result in aggregated load curve that matches the aggregated supply curve. Figure 4 illustrates the concept of SDEN as a cyber-physical system, and we explain it further as follows.

Consider the following relevant (physical) infrastructure of the industrial plant.

(1) different processing machines that perform different types of jobs with distinct energy demand profiles;
(2) renewable energy sources with zero-marginal cost (e.g., solar PVs) and battery storage;
(3) connection to the main grid where energy has a non-zero cost;
(4) sensors measuring the attributes of relevant processes that are connected to a data network.

Overall, the cyber-physical system depicted in Figure 4 contributes to a seamless integration between energy supply and demand, enhancing energy management. It enables real-time data collection, analysis, and decision-making to allocate energy efficiently in line with the dynamic needs of industrial processes. The interconnected structure of the physical and cyber layers ensures optimized energy use, leading to reduced energy waste, enhanced operational efficiency, and cost savings within industrial applications.

From previous points, the SDEN elements can be defined based on their roles in the industrial cyber-physical system under consideration. Table 1 presents the elements of the SDEN (cyber domain) together with their respective physical entities. For example, an industrial machine that performs a given job is virtualized as an SDEN client; this is possible because such a machine demands electric energy to perform that given job, which, in turn, offers some flexibility in terms of when it should be accomplished.

**Table 1:** Elements of the proposed industrial SDEN

Cyber element	Physical entity	Examples
SDEN Client	Flexible industrial load	Machines or Jobs
SDEN Source	Energy producers	Solar PVs, windmills
SDEN Storage	Energy storage	Batteries
SDEN Server	Computing device(s)	Software in cloud/edge

The virtualization process is carried out by a technique called PEM (Nardelli et al.^6^; Espinosa et al.^27^; Khurram et al.).^39^ The main idea is to map the electric power required by a given device/machine/process to perform a given task/job into discrete energy packets, which is simply defined as the average energy consumed during a predetermined duration divided by the unit packet size. As an illustration, let a unit packet size be 1 Wh and the duration be 1 min; then, a machine that consumes 10 Wh in 1 min will be virtualized as 10 energy packets.

After the loads are virtualized in packets, different SDEN clients request to the SDEN server to use the shared energy resources. The server needs to sense, based in the network connections, the states of the storage units, and the weather in order to decide the scheduling. The scheduling can be solved as a variation of the energy-aware job shop optimization problem. The scheduling task is to find the (sub)optimal resource allocation of jobs in terms of energy self-sufficiency (i.e., minimizing the use of energy from the grid) considering the SDEN source enables N[t] packets to be used at the same time slot *t*; the storage unit may be charged or discharged giving its state S[t] where $S\in \left[0,S_{\max}\right]$. The challenges to solve this combinatorial optimization problem and a potential approach to solve such job-shop scheduling problems will be presented in the sections that follows.

### Machine-type communications and (beyond) 5G-enabled mobile edge computing

Industrial DSM requires support from a communication network that is designed to support machine-type communications (MTC) in different regimes, namely reliable massive connectivity (to support the SDEN operation) and ultra-reliable low latency (to guarantee the physical operation of the industrial microgrid) including adaptive protection devices (Gutierrez-Rojas et al.).^40^ As described in Nardelli et al.,^28^ the 5G-enabled MTC for “energy vertical” employs private communication networks via local licenses together with mobile edge computing capabilities so that computations are closer to the end applications to decrease the communication latency (Narayanan et al.).^41^ However, most studies focus either on applications in household demand response or automation of distribution grids in general, not considering the specific challenges of industrial microgrids; one interesting exception partly related to this proposal is found in Peng et al.^42^

## Explainable and trustworthy AI-based networked sensing for energy packets

As we discussing in previous section, explainable networked sensing and SDEN work in concert to enhance transparency and understanding of energy management processes. It transforms data into meaningful information by identifying patterns and trends. This context makes the data more understandable and actionable. In the following subsection, the enablers of explainable network sensing are reviewed.

### Artificial intelligence and machine learning

AI can be defined as “the ability of a computer or machine to perform tasks that typically require human intelligence, such as visual perception, speech recognition, decision-making, and language translation” (Russell and Norvig).^43^ Such intelligent AI machines or systems are capable of learning from data, recognizing patterns, and making decisions with minimal human interventions. Recently, AI has grown tremendously, leading to numerous breakthroughs and developments in the fields of robotics, ML, computer vision, natural language processing, robotics, etc. (Goodfellow et al.).^44^ Today, the most popular subfield of AI is *ML*, defined succinctly by Tom Mitchell as follows: “A computer program is set to *learn* from an experience *E* with respect to some task *T* and some performance measure *P* if its performance on *T* as measured by *P* improves with experience *E*.” (Russell and Norvig).^43^ ML can be considered to be as a combination of three components: (1) data, (2) a model or hypothesis space, and (3) a loss function that measures the quality of a hypothesis map (Jung).^45^

### Networked data

The server makes decisions that are highly dependent on the underlying networked data. So, data quality and availability play crucial roles in guaranteeing the transparency, comparability, and reproducibility of models/strategies used for decision-making. Examples of this type of industrial applications are modern power systems that have a big share of renewable energy and, empowered by ICT, use fully networked models to achieve intelligent management goals (Zhang et al.^46^; Butt et al.)^47^; this is in contrast with traditional power models that mainly focus on physical properties such as power balancing and load on the grid (Schavemaker and Van der Sluis)^48^ or market models that concern themselves with increasingly liberalized electricity markets (Ventosa et al.^49^; Pantoja-Robayo^50^; Kühnlenz et al.).^51^ In these networks, a various numbers of heterogeneous communication nodes or entities are defined, such as supply sources (conventional centralized generation plants or distributed wind turbines/solar panels using renewable resources), transshipment junctions, remote center servers, production plants, residential users, electric vehicles, and connected houses, etc. These nodes are digitally addressable and equipped with various smart meters, sensors, and/or processors, which can be used for data collection, transmission, storing, and analysis in a fast and comprehensive way (Nygard et al.^52^; Medjroubi et al.^53^; Zhang et al.^46^; Daki et al.).^54^

Explainable networked sensing is crucial in modern industrial systems that use fully networked models to achieve intelligent management goals. These systems have a variety of digitally addressable and equipped communication nodes that collect, transmit, store, and analyze large-volume temporal and spatial data in a fast and comprehensive way. These data are crucial for decision-making, with data quality and availability important for guaranteeing transparency, comparability, and reproducibility of models/strategies. To efficiently manage these data, efficient data management systems must be built with high velocity, security, storage capacity, advanced data analytics, and fast decision-making capabilities.

### Trustworthy AI

As mentioned previously, smart grids in power systems, in general, require extensive data collection and processing. Smart meters are used to report, measure, and monitor power quality metrics, flows, and loading conditions that are used as the source for data analysis (Antonov et al.).^55^ Importantly, ML methods employed during energy provision should fulfill certain qualitative requirements, since potentially substantial risks may emerge from faulty, or biased models (Xu et al.).^56^ This is necessary to ensure that professionals using AI systems are able to understand and evaluate the performance of models and therefore the trustworthiness of the model. Given the crucial nature of energy provision, it is proposed here that the Guidelines for Trustworthy AI (the Guidelines) should be utilized as Guidance when choosing and making the AI system. The Guidelines were made by the High-Level Expert Group on AI (HiLeg) that was established by the European Commission with the vision to,

(1) increase public and private investments in AI to boost its uptake,
(2) prepare for socio-economic changes, and,
(3) ensure an appropriate ethical and legal framework to strengthen European values (High-Level Expert Group on AI).^57^

The Guidelines proposes that Trustworthy AI must always be lawful, ethical, and robust (High-Level Expert Group on AI).^57^ It then sets out seven requirements for AI: (1) Human agency and oversight, (2) Technical robustness and safety, (3) Privacy and data governance, (4) Transparency, (5) Diversity, non-discrimination, and fairness, (6) Societal and environmental well-being, and (7) Accountability (High-Level Expert Group on AI).^57^ It is noteworthy that the requirements do not only consist of technical requirements, but also organizational measures, such as impact assessments for societal impacts and environmental impacts.

The first requirement—“human agency and oversight”—refers to the necessity of AI systems to support human autonomy and decision-making rather than leaving decisions to a black box model. Human agency refers to the capability of a human to intervene into the AI system. While the Guidelines lay out different approaches, it is important to note that the human interacting with the AI system should be able to exercise control, not only during the development but also during the use of the system. It is important to ensure that a human can oversee the operations of the AI system and intervene into the decision-cycles, and also override system decisions where they are considered to be faulty (High-Level Expert Group on AI).^57^

“Technical robustness and safety” inherently aims at the prevention of harm. The model should be reasonably resilient against potential attacks, but nevertheless, might comprise faulty decision-making through over-reliance on outliers. Under this requirement, HiLeg also summarizes the need for accuracy, reliability, and reproducibility (High-Level Expert Group on AI).^57^

The requirement of “privacy and data governance” refers to the necessity to safeguard one’s privacy while processing data together with the applicable legal requirements. Importantly, the requirement includes the entire life cycle of the data and the AI system. It is necessary to plan the whole AI system to be applied with “privacy and data governance” in mind and to include crucial requirements such as data minimization, data quality, and storage limitation. Before the model is trained with real-life data, it is necessary to define which data may be collected and processed for which purpose and under which conditions. It is necessary to conduct a “Data Protection Impact Assessment” that inherently necessitates detailed planning of the future processes together with the mitigation of potential risks. “Transparency” as a requirement is closely related to privacy and data governance as well as human oversight. Transparency refers to the data, system, and business model and requires that all operations are appropriately documented. It also requires explainability (High-Level Expert Group on AI).^57^In the case of AI tools within energy provision, there are circumstances where explainability is required by law. Where personal data are collected, the necessity for explanation stems from the General Data Protection Regulation (GDPR) that regulates the use of personal data within the European Union and seeks to harmonize the protection fundamental rights and freedoms, particularly the rights to data protection and privacy, within the European Union. The GDPR mandates the right not to be subjected to automated decision-making in Article 22 of the GDPR. Articles 13 (2) (f), 14 (2) (g), and 15 (1) (h), read in combination with Article 22 of the GDPR, require meaningful information about the logic involved in automated decision-making and the significance of envisaged consequences. Recital 71 of the GDPR, in referring to this Article 22, requires suitable safeguards such as the right to specific information to the data subject, the right to obtain human intervention, and the right to obtain an explanation of the decision reached. While the right to explanation under the GDPR has been disputed in literature (Sandra Wachter),^58^ it is important to take this into account when it comes to the trustworthiness of a system.

“Diversity, non-discrimination, and fairness” require the systematic planning and analysis of the potential outputs of the AI system. Unfair biases shall be circumvented, and accessibility and universal design shall be taken into account to ensure user friendliness. Stakeholders shall be involved in the process to ensure that feedback can be gathered in order to circumvent bias and hurdles regarding accessibility (High-Level Expert Group on AI).^57^ Importantly, when it comes to the notion of fairness, there is no consensus about its meaning (Wrigley).^59^ However, the minimal requirement that can be derived from the Guidelines is the avoidance of unfair bias.

“Societal and environmental well-being” shall be achieved by ensuring critical examination of the use of resources and energy, and by abstaining from harmful choices. Further, it is requested that social impacts are evaluated and good choices are made (High-Level Expert Group on AI).^57^ Therein, the requirement of “accountability” requires the possibility for auditability, ultimately also giving rise to efficient monitoring of the AI system and its output. It is important to note that in any case, these guidelines should be followed; as a matter of fact, many of these requirements are also found in other legal framework, particularly in the GDPR. However, the GDPR might not always be applicable, as it only applies to the treatment of personal data, as exposed in High-Level Expert Group on AI.^57^

### Networked sensing for informed decision-making in cyber-physical systems

Following the conceptualization introduced in Nardelli,^38^ networked sensing refers to the data acquisition and processing steps that serve to perform informed decision-making, which is then employed to determine actions in the cyber-physical system in consideration. In this way, networked sensing builds a *structure of awareness* partly from the sensors’ data that create a dynamic “virtual map” (or even a digital twin) of the physical problem to be solved. From this virtual map, decisions based on information (i.e., data that are capable of decreasing uncertainty) can be made. Decision-makers can be either machines (computers) or humans; the decision can be centralized, decentralized, or distributed. Once a decision is taken, then agent(s) can act following the *structure of action* of the cyber-physical system. In the SDEN case under consideration, the focus is on automatic decision-making by a central element—the SDEN server—which processes the data based on an AI engine. The SDEN server as a software-defined element can be implemented in hardware in different ways from one local computer to a cloud computing platform, from a mobile edge computer to a federated implementation based on big data over networks (Sarcheshmehpour et al.^60^; Narayanan et al.).^41^ Regardless of the selected implementation, the outcomes of the AI engine need to offer to a rationale for its outcomes: the decision must be explainable. This is important because in industrial environments the “black-box approach” may not suffice (Ahmed et al.).^61^ In the specific case we have, the explanation would be determined by logical relations that should have a semantic description like “job 1 it to be performed by machine A using setting X starting at 13:03 because the sun was shining and the battery is fully charged; this was the job with the longest duration among the others that requested.”

The explainability of automated decision-making systems built on predictive methods is crucial for the acceptance and success of the SDEN, because it can optimally explain the proposed energy-centric schedule of the ICPS activities to the different relevant actors; this is necessary condition to build trust in the proposed solution. The main focus of the explainable AI optimization is to solve a variation of the multi-objective (energy-aware) flexible job-shop scheduling optimization problem with focus on matching supply and demand of electricity, considering the specifics of the sequential nature of the industrial process (Wang et al.^7^; Mota et al.).^8^ in a distributed manner. This is in contrast to the typical centralized approach based on non-linear optimization or metaheuristics mostly using a static or a probabilistic generated dataset. We now show how a flexible job-shop scheduling optimization problem could be solved in this distributed manner through collective learning, whereas the explainable AI engine that will power the SDEN will be implemented in the future.

## Resource allocation for industrial microgrids

In this section, we discuss the idea of packetized energy management that was discussed in the previous section. We first show how the energy consumed by machines in an industrial manufacturing process can be packetized and managed. Then, we show how we can perform additional optimization of the job shop schedules using a combinatorial optimization approach based on the coordination of the energy packets required by the machines.

### Packetization

We consider the scheduling problem in an energy-intensive additive manufacturing (3D printing) process that was introduced in Karimi et al.^9^ and further explored in Karimi and Kwon.^10^ The numerical case study is the same as in Karimi and Kwon^10^ where there are three fused deposition modeling (FDM) machines that can operate in parallel and ten jobs to be accomplished. The stages of FDM are pre-heating, printing, and bed cooling (as illustrated in the case study). The ten jobs to be performed are of four different types: three of types 1 and 2 and two of types 3 and 4; the energy demand in these processes are given in Table 2. Table 2 also includes the number of energy packets considering a reference unit of 1 Wh (equaling 60 W-min) and 2-min duration.

**Table 2:** Energy demand for the three different machines to perform each of the four different jobs

Job types	Stages	Machine 1		Machine 2		Machine 3	
		Power (W)	Duration (min)	Power (W)	Duration (min)	Power (W)	Duration (min)
1	1	364	2	352	12	371	8
	2	196	50	300	24	302	34
	3	42	6	43	12	43	12
2	1	357	2	373	10	372	8
	2	203	62	320	26	379	38
	3	43	6	48	14	44	12
3	1	370	6	371	14	372	8
	2	242	50	338	22	290	32
	3	42	10	43	14	43	12
4	1	354	2	371	10	372	8
	2	201	28	334	6	303	14
	3	48	6	45	12	46	12

Adapted from Karimi et al.^9^

The proposed SDEN works in this manner. The machines are Energy Clients that request Energy Packets to perform the jobs. At the beginning of each day (e.g., 7 a.m.), the machines connect to the data network to send their requests and their individual flexibility. For example, Machine 1 requests permission to perform three jobs of types 1 and one of type 3 from 8 a.m. to 3 p.m. After collecting the requests, the Energy Server creates a virtual map of the energy network estimating the energy availability from the Energy Source throughout the day (e.g., from 8 a.m. to 4 p.m.). The Energy Storage is also considered to provide an additional flexibility by acting as a buffer, storing energy when the supply is greater than the demand, and compensating the energy deficit otherwise. The Energy Server then processes the data in a decision-making process that will allocate the resources to the Energy Clients, indicating the accepted jobs that they will perform and the appropriate timing. In this way, the Energy Server performs the packetized energy management by multiplexing the energy packets following the individual requirements and feasible plan to accomplish the tasks of the day.

**Figure 5:**
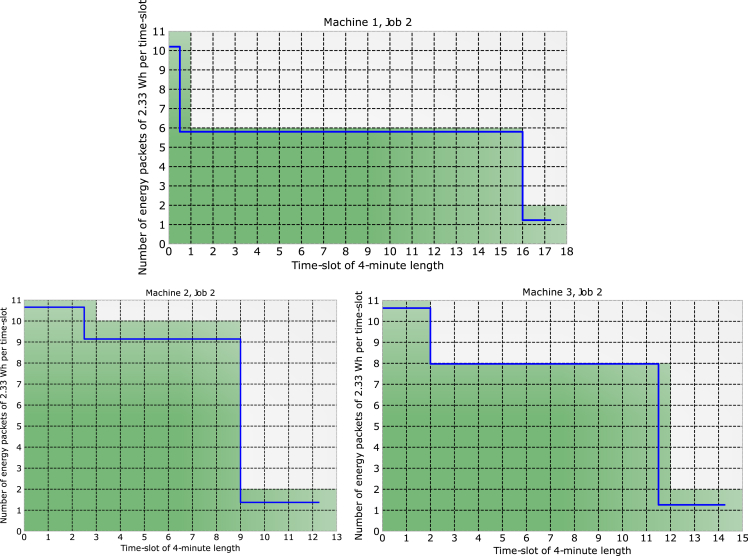
Machines 1, 2, and 3 performing job 2 using the same energy packet sizes

**Figure 6:**
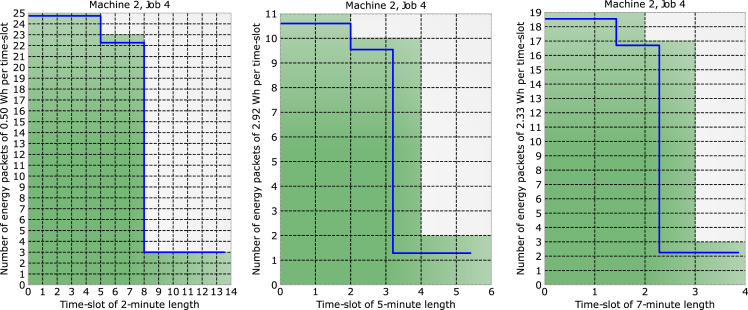
Machine 2 performing the same job with 3 different energy packet sizes

The principle of the proposed SDEN is shown in Figure 5. It shows the power plot of 3 different machines as they perform the job # 2. Each energy packet that is required to perform such a job is shown in green. The time slot chosen in this case was 4-min length on the x−axis resulting in 2.33 Wh per time slot (each green square). Machine 1 required 105 energy packets, machine 2, 101 packets, and machine 3, 108 packets. The number of packages required by each machine depends on the job they perform and the packet size defined by the energy server. The size of the packets given the job performed by a machine can be seen in Figure 6. Depending on the size of each packet, for the same job, a machine can require less or more packets. In 6, the job # 4 is performed by machine 2, when the energy packets have a size of 2.33 Wh (7-min length); the machine requires 58 packets for a total amount of consumed energy of 135.14 Wh. When the energy packets have a size of 2.92 Wh (5-min length), the machine requires 46 packets for a total consumption of 134.32 Wh. Finally, when the energy packets have a size of 0.5 Wh (2-min length), the machine requires 212 packets for a total of 106 Wh. Packet sizing is a crucial task conducted by the energy server since it can lead to massive energy savings depending on various factors such as machine, job, and time frame. Ideally, the energy server aims for energy packets that are smaller so that they fit the curve of each machine better, but, in some cases, this is not possible due to the load demand.

### Energy-centric flexible job-shop scheduling optimization

We will now use job-shop scheduling to size the packets for the packetized management.

#### Background

The job-shop scheduling problem (JSSP) is one of the most well-known combinatorial optimization problems, and it has been extensively studied (Xie et al.^62^; Xiong et al.).^63^ The JSSP considers that *n* different jobs—$J=\left\{J_{1},J_{2},\ldots ,J_{n}\right\}$—have to be scheduled on *m* machines. Each job $J_{i}$ consists of a set of tasks—$O_{i}=\left\{O_{1i},O_{2i},\ldots ,O_{ki}\right\}$—that must be performed in a given sequence. In the traditional JSSP, each operation requires a specific machine for processing, and only one operation in a job can be processed at a time. However, in *flexible* job-shop scheduling (FJSSP), some, or all, operations can be processed on any machine of a given set. In general, the JSSP aims to schedule the tasks on the machines while minimizing the time needed, cost required, or other production efficiency measures. Solving the JSSP is not trivial and is, in fact, NP-hard, because the solution space is huge with the number of possible job-machine combinations being of the order of (*n*!)^*m*^ (Para et al.).^64^ To solve the JSSP and FJSSP, both exact (deterministic) methods such as mixed-integer linear programming (MILP) (Xie et al.)^62^ as well as heuristics and metaheuristics such as genetic algorithms (Xie et al.^62^; Xu et al.)^65^ and evolutionary algorithms (Gao et al.^66^; Xie et al.)^62^ have been attempted, while, more recently, deep learning has also been employed (Cunha et al.).^67^

In recent years, reductions in energy consumption and maximization of energy efficiencies have become important objectives for industries. By exploiting the flexibility available in some manufacturing processes and machines, e.g., by machine selection, job sequencing, distributed scheduling, decision-making by switching off/on the machines, or worker availability, energy consumption can be effectively decreased (Zhang et al.^68^; Meng et al.^69^^,^^70^; Gong et al.^71^; Para et al.).^64^

#### Problem model

The energy-aware FJSSP problem can be mathematically stated as follows. Following the MILP formulations given in Özgüven et al.^72^ and Meng et al.,^69^ consider a set of *m* machines $M=\left\{M_{1},M_{2},\ldots ,M_{m}\right\}$, a set of *n* jobs $J=\left\{J_{1},J_{2},\ldots ,J_{n}\right\}$, and a set of *r* operations for each job $J_{i}$, $O=\left\{O_{i1},O_{i2},\ldots ,O_{ir}\right\}$. Each operation $O_{ij}$ can be processed by employing a subset of eligible machines $M_{ij}\subseteq M$. Further, assume that the time and energy required for an operation $O_{ij}$ to be completed on a machine *k* is given by $t_{ijk}$ and $E_{ijk}$, respectively, ∀i=1,…,n, ∀j=1,…,r, and ∀k=1,…,m. The total energy consumed by all the jobs is given by $E=\sum_{i,j,k}E_{ijk}$. We can also make the following typical assumptions:

(1) All the jobs and machines are available in the beginning, i.e., time 0.
(2) The operations of different jobs are independent.
(3) All the operations of the same job must be processed in the given order.
(4) All the machines process only one operation at a time, and every operation can be processed on only one machine.
(5) All machining data such as processing time, processing power, etc., are known in advance.
(6) An operation cannot be interrupted once it is started.

The energy-centric FJSSP aims to choose the appropriate machine for each operation and determine the job sequences for every machine with the objective of minimizing the total energy consumption (or, some other energy-related objective, e.g., minimization of carbon footprint, CO_2_ emissions, etc.). Then, we have the following decision variables. A binary decision variable $X_{ijk}$ is used for the machine selection; $X_{ijk}=§1$, if a machine *k* is selected for an operation $O_{ij}$ and 0, otherwise. A binary variable $Y_{iji^{\prime }j^{\prime }k}$ can be used to verify the sequence of operations; $Y_{iji^{\prime }j^{\prime }k}=1$ if an operation $O_{ij}$ precedes operation $O_{i^{\prime }j^{\prime }}$ on machine *k* and 0, otherwise. Depending on the problem formulation, we can also have the completion time of a job *i*, $C_{i}$, and maximum completion time over all jobs (makespan), $C_{\max}$.

The most common objective in FJSSP models studied so far is to minimize the makespan $C_{\max}$. However, in energy-aware FJSSP, which emphasizes energy savings, the objective is often to minimize the total energy consumption $E=\sum_{i,j,k}E_{ijk}$. The constraints in the problem are set to meet the abovementioned assumptions. Excellent examples of MILP formulations for the FJSSP as well as energy-aware FJSSP are given in detail in Özgüven et al.^72^ and Meng et al.,^69^ respectively. The methods applied to solve the energy-aware variations of the FJSSP are similar to the approaches to the JSSP and FJSSP, comprising a mix of both exact methods and heuristics, for example, MILP (Zhang et al.^68^; Meng et al.),^69^^,^^70^ heuristics and metaheuristics such as genetic algorithms (May et al.^73^; Para et al.),^64^ particle swarm optimization (Tang et al.),^74^ etc.

#### Collective-intelligence-based approach

In this paper, we propose that collective intelligence and collective learning principles can also be used to solve JSSP, FJSSP, and its energy-centric variants. Collective intelligence refers to the aggregated shared intelligence that results from interactions and coordinated actions among distributed agents (human or software or both) (Narayanan et al.).^75^ Since collective-intelligence methods can achieve effective coordination and scalability through parallel processing, they are especially useful for industrial applications in which machines can operate at the same time, in parallel. Traditionally, collective intelligence has been realized by swarm intelligence and bio-inspired computing methods such as particle swarm optimization, artificial bee colony algorithm, and ant colony optimization. Collective learning refers to the production of knowledge and collective intelligence as a result of dynamic and evolutionary interactive processes among the different agents, typically human and software agents, in the network or system (Garavan and Carbery).^76^ Recently, several collective-learning approaches have been proposed for coordination and combinatorial optimization problems (Yu et al.^77^; Hao et al.^78^; Jin and Ma^79^; Pournaras et al.).^80^ Among them, Pournaras et al. proposed a general-purpose decentralized collective learning algorithm and tool called Economic Planning and Optimized Selections (I-EPOS) that has a fully decentralized approach for coordinated multi-objective decision-making in multi-agent systems (Pournaras et al.).^80^

We will now illustrate the potential applicability of I-EPOS to job-shop scheduling problems using packetized energy management by modeling a simple scenario. Assume that there are 3 machines, $M=\left\{m_{1},m_{2},m_{3}\right\}$, all of which are designed to manufacture an identical product. They manufacture this product by performing a job that has 10 identical operations that do not need to be performed in sequence. In other words, all the machines can perform the *same* operations to manufacture the product. However, the energy consumption and time requirements for each operation are different for the different machines. This situation mirrors the case where a company may have a mix of different machines, e.g., old and new models, for performing the same tasks. It is difficult to obtain real measured industrial machine consumption data due to proprietary reasons for many jobs. Further, in this paper, our objective is to illustrate the potential of collective learning to solve energy-aware FJSSP problems. Hence, the settings for the three machines were generated randomly and are listed in Table 3. Further, we assume that this manufacturing process is powered by solar energy, and sufficient renewable energy to run the three machines together in parallel is available for only 6 h. Therefore, we have the additional constraint that a machine can only operate up to 6 h. Our objective is to minimize the total energy consumed and time required to manufacture a single product.

**Table 3:** Energy demand for the three different machines to perform the 10 operations required to produce a product

Operations	Machine 1		Machine 2		Machine 3	
	Energy (kWh)	Duration (min)	Energy (kWh)	Duration (min)	Energy (kWh)	Duration (min)
1	230.74	173	228.08	89	348,82	78
2	390.26	48	284.22	141	30.11	73
3	59.14	118	9.39	59	333.38	10
4	319.96	68	308.82	40	335.32	149
5	71.68	104	306.05	88	105.19	116
6	472.33	10	308.47	175	64.46	80
7	260.92	22	471.87	89	157.71	176
8	207.33	37	340.91	82	181.86	83
9	132.28	88	179.75	166	285.1	100
10	387.12	71	218.52	26	219.3	178

The collective learning algorithm I-EPOS was used to solve this problem by combining it with a plan-generation algorithm in the following manner. For each machine, we generated a finite set of possible plans. Each plan corresponded to a set of operations that were associated with the energy consumed. These plans were generated such that the time taken is always less than 360 min. For example, one possible plan for a machine, $m_{1}$, could be 831.27:{1,7,8,9}, implying that $m_{1}$ performs operations 1, 7, 8, and 9 with an energy consumption of 831.27 kwh. These plans were then combined using I-EPOS such that all 10 operations were performed by the 3 machines with minimum energy consumption. There were 50 iterations in the I-EPOS setup, and 25 simulation rounds were conducted.

If there is unlimited energy supply (and therefore, unlimited time), each machine can perform these operations independently without any coordination among each other. In this case, machines 1, 2, and 3 will consume 2531.76 kWh, 2656.08 kWh, and 2061.25 kWh, respectively, taking a time of 739 (12 h, 19 min), 955 (15 h, 55 min), and 1043 min (17 h, 23 min), respectively. However, when I-EPOS was used to coordinate the machines and determine their parallel schedules, the following results were obtained. The following set of operations were chosen for machines 1, 2, and 3, respectively: [1,9], [3,4,5,7,10], and [2,6,8]. Moreover, by conducting parallel operations in this manner, the three machines collectively and collaboratively performed the tasks within 5 h 2 min (261, 302, and 236 min, respectively) with a fully renewable energy consumption of 1954.1 kWh. This represents a 5% decrease in energy consumption as compared to the uncoordinated case.

## Conclusions, lessons learned, and future directions

This paper presented the three most relevant enabling technologies for industrial energy management—software defined energy network, explainable trustworthy AI-based network sensing, and resource allocation methods—that can provide significant benefits to industrial energy management, including improved efficiency considering the renewable sources. Software-defined energy network is the high-level conceptual architecture comprising cyber-physical operations of the grid based on a client-server relationship where agents (machines) request the use of energy resources and a server (computer) either grants it or not, and if granted, schedules it. Explainable trustworthy AI for networking sensing enhances the transparency and accountability of energy management systems in general and will be a key designing principle of the energy server decision method, making it more trustworthy and easy to understand. Packetized energy management is the technique to virtualize the physical energy system and enable better planning and execution of an energy-centric industrial scheduling, thereby improving its responsiveness and adaptability.

Further, using numerical examples, we show how the problem can be formulated in industrial scenarios. We demonstrate energy packetization using the energy data that we obtained from an industrial manufacturing process (3D printing) and show the trade-offs between the time granularity and the size-unit of the packets. These parameters are related to the application of solutions in the cyber-domain to those in the physical domain, which are defined at the Energy Client level. In addition, we also demonstrate the potential of using collective learning to solve the scheduling optimization problem of the Energy Server.

In the future, we plan to combine explainable AI-based methods with the PEM methods and test them on simple cases with data acquired in actual industrial deployments or via more realistic simulation of industrial plants with energy generation and storage capabilities. Other possible future directions of the work could include further testing and optimization of the X-SDEN concept, with a focus on improving energy efficiency and reducing the reliance on non-renewable sources. This could involve refining the design of the software-defined energy network and resource allocation methods, as well as developing new explainable AI techniques for networking sensing. Additionally, real-world testing of the method in industrial settings, or through realistic simulations, could provide valuable insights into its performance and potential for widespread adoption. Finally, exploring the use of alternative energy sources and developing strategies for integrating them into the X-SDEN framework could be another promising area for future research.
